# Hypoxic induction of vasculogenic mimicry in hepatocellular carcinoma: role of HIF-1 α, RhoA/ROCK and Rac1/PAK signaling

**DOI:** 10.1186/s12885-019-6501-8

**Published:** 2020-01-13

**Authors:** Ji-Gang Zhang, He-Ming Zhou, Xue Zhang, Wan Mu, Juan-Ni Hu, Gao-Lin Liu, Qin Li

**Affiliations:** Department of Clinical Pharmacy, Shanghai General Hospital, Shanghai Jiao Tong University School of medicine, No.100 Haining Road, Shanghai, 200080 People’s Republic of China

**Keywords:** HIF-1α, RhoA/ROCK, Rac1/PAK, Vimentin;Vasculogenic mimicry

## Abstract

**Background:**

Vasculogenic mimicry (VM), defined as a capability of aggressive tumor Cells to mimic embryonic vasculogenic networks, caused poor prognosis in hepatocellular carcinoma (HCC). Rho kinases (ROCK), p21-activated kinase (PAK), hypoxia or epithelial-mesenchymal transition (EMT) contributed to the VM potential. However, the details underlying these biological behaviors have not been completely elucidated.

**Methods:**

Kaplan-Meier analysis was conducted to predict relationship with hypoxia Inducible factor (HIF-1α), EMT related markers: Vimentin and patient prognosis. CD34/periodic acid-Schiff (PAS) double staining was examined to differentiate VM-positive (VM+) and VM-negative (VM-) samples. Cells were cultured under controlled hypoxic environments (1% O2) or normoxic conditions. The effect of hypoxia on RhoA/ROCK, Rac1/PAK and EMT were evaluated by real time-qPCR and western blot. HIF-1α small interfering RNA (siRNA), overexpressed or short hairpin RNA (shRNA) of ROCK and kinase inhibitors were used to explore the effect of HIF-1α, RhoA/ROCK, Rac1/PAK and Vimentin on VM.

**Results:**

HIF-1α or Vimentin was upregulated in VM+ HCC tissues, compared to non-cancerous tissues (*P* < 0.01), and patients with high expression of HIF-1α or Vimentin had worse prognosis (*P* < 0.001). We showed hypoxia induced RhoA/ROCK and Rac1/PAK signaling transduction, and EMT could be repressed by HIF-1α siRNA. Notably, RhoA/ROCK or Rac1/PAK stabilized HIF-1α in hypoxia, whereas HIF-1α did not significantly altered RhoA/ROCK or Rac1/PAK signaling in hypoxia. Moreover, we found distinct roles of ROCK1, ROCK2 and PAK in regulating Vimentin phosphorylation.

**Conclusions:**

RhoA/ROCK and Rac/PAK signaling played crucial roles in hypoxia-induced VM via Ser72 and Ser56 Vimentin phosphorylation in HCC.

## Background

Hepatocellular carcinoma (HCC) is a prevalent malignancy, of which incidence and mortality rates have remained at a higher level [1]. While there have been some advanced strategy of diagnosis and treatment applied to clinical practice [2], the dismal outcome and poor median survival of patients have been largely unchanged for decades. Tumors required an endothelial-independent blood supply to break through the limitations of oxygen and essential nutrients by vasculogenic mimicry (VM), which was first described by Maniotis et al. [3]. Our previous reports have discovered that VM and mosaic vessels are involved in HCC and played a critical role in promoting cancer metastasis, invasion, which led to poor prognosis [4, 5]. Therefore, it is indispensable to investigate more about pathological mechanism involved.

Hypoxia is one of the most ordinary phenomenon in tumor microenvironment on account of the abundant oxygen and energy consumption [6]. Several studies have shown the stimulative effect on hypoxia-inducible factor-1α (HIF-1α) activity, which played a critical role in regulating the adaption of carcinomas to hypoxia [7], on VM formation [5, 8, 9]. The p21-activated kinases (PAKs) belong to a family of serine/threonine kinases that act as effectors of the Rho-related GTPases like Rac and Cdc42 [10]. PAK phosphorylate a series of proteins controlling cytoskeletal remodeling, cell survival and motility as well. Given the bulk of hypoxic regions in solid tumors, elucidating links between PAK and HIF-1α in hypoxic cancer cells may further unveil details in mechanisms of cancer development. Rho kinase (ROCK) [11], was associated with cell migration, invasion, cell-cell adhesion [12–14]. Our previous report mentioned that ROCK plays an important mediated role in the process of cancer cell VM formation [4]. Recently, a great deal of researches have applied RNAi technology to independently disrupt each paralog of ROCK (ROCK1 and ROCK2) in vitro and have unveiled unique roles for either ROCK1 or ROCK2 in the control of actin-cytoskeleton dynamics and cell morphogenesis, migration, cell fate decisions, and extracellular matrix assembly [15, 16]. Therefore, HIF-1α, ROCK isoforms and PAK seemed to be potential molecular targets for the investigation of VM research.

During epithelial-mesenchymal transition (EMT), epithelial cells gradually dedifferentiate, lose cell polarity with epithelial surface markers degeneration, turning to expressing mesenchymal markers, displaying phenotypic alterations, similar to VM process, which mimicked endothelial cells possessing the capability of tube formation. Recently, EMT has been considered as a mechanism leading to VM [17, 18]. The molecular transitions for EMT are the upregulation of mesenchymal components, like Vimentin and N-cadherin and the decrease of E-cadherin expression, which belongs to epithelial cell adhesion molecules [19].

RhoA and Rac pathways are the two principle signaling pathways which implicated in both mechanosensing and the regulation of the Vimentin network. ROCK or PAK was involved in Vimentin phosphorylation and rearrangement [20, 21]. Nevertheless, the mystery of molecular mechanism between HIF-1α, RhoA/ROCK, Rac1/PAK and Vimentin have not been fully unveiled.

Here, we are trying to investigate RhoA/ROCK and Rac1/PAK expression triggered by hypoxia in order to explore whether HIF-1α activation is significant for VM formation as well as inducing the EMT phenotype. As far as we know, this research is the first study elaborating the relationship between HIF-1α, RhoA/ROCK, Rac1/PAK and EMT in HCC exposed to hypoxia.

## Methods

### Collection of patient samples

In total, 80 pairs of HCC tissues and adjacent non-tumorous liver tissues were purchased from ShGnghGi Outdo Biotech Company (China). Three patients were ruled out for lacking completed clinical and follow-up data. Written informed consent was obtained from all subjects, and the study was approved and supervised by the Ethics Committee of the Shanghai General Hospital, Shanghai Jiao Tong University School of Medicine. The completely pathological and clinical features of all the patients were shown and stored in Table 1.

**Table 1:** Associations between HIF-1α, Vimentin and clinico-pathological characteristics in HCC

Variant	No. of patients	HIF-1α		*p*-value	Vimentin		*p*-value
		Low(%)	High(%)		Low(%)	High(%)	
Cases	77	34(44.2)	43(55.8)		27(35.1)	50(64.9)	
Age (years)							
≤ 60	62	28(45.2)	34(54.8)	0.718^a^	21(33.9)	41(66.1)	0.655^a^
> 60	15	6(40.0)	9(60.0)		6(40.0)	9(60.0)	
Gender							
Male	63	27(42.9)	36(57.1)	0.626^a^	19(30.2)	44(69.8)	0.056^a^
Female	14	7(50.0)	7(50.0)		8(57.1)	6(42.9)	
Tumor size (cm)							
≤ 3	35	18(51.4)	17(48.6)	0.241^a^	15(42.9)	20(57.1)	0.191^a^
> 3	42	16(38.1)	26(61.9)		12(28.6)	30(71.4)	
Clinical stage							
I/II	42	23(54.8)	19(45.2)	0.065^a^	20(47.6)	22(52.4)	0.011^a^*
III/IV	35	11(31.4)	24(68.6)		7(20.0)	28(80.0)	
Invasion depth							
T1 + T2	40	16(40.0)	24(60.0)	0.445^a^	17(42.5)	23(57.5)	0.155^a^
T3 + T4	37	18(48.6)	19(51.4)		10(27.0)	27(73.0)	
Lymph nodes metastasis							
N0 (negative)	46	25(54.3)	21(45.7)	0.028^a^*	22(47.8)	24(52.2)	0.016^a^*
N1 (positive)	31	9(29.0)	22(71.0)		7(21.2)	24(78.8)	
Distant metastasis							
M0 (absent)	72	33(45.8)	39(54.2)	0.510^b^	25(34.7)	47(65.3)	0.811^b^
M1 (present)	5	1(20.0)	4(80.0)		2(40.0)	3(60.0)	
VM							
Negative	50	27(54.0)	23(46.0)	0.018 ^a^*	22(44.0)	28(56.0)	0.025^a^*
Positive	27	7(25.9)	20(74.1)		5(18.5)	22(81.5)	

^a^ Chi-square test

^b^ Fisher’s exact test

*P < 0.05 indicates a significant association among the variables

### Materials; antibodies and cell lines

Materials, chemical agents and antibodies applied in this research included Matrigel (BD Biosciences); C3 transferase and Y27632 (Sigma-Aldrich); NSC23766 and IPA (Selleck); cell culture media (DMEM); fetal bovine serum (FBS) and antibiotics (Gibco); Furthermore, the information of antibodies was listed in Additional file 1: Table S1.

MHCC97H cell lines were obtained from the Liver Cancer Institute, Zhongshan Hospital, Fudan University (Shanghai, China). Cell lines were cultured in DMEM supplemented with 10% FBS and 1% antibiotics, maintained at 37 °C in a humidified atmosphere of 5% CO_2_, or under controlled hypoxic environments (1% O_2_, 5%CO_2_ and 94%N_2_.)

### RNA interference (RNAi)

MHCC97H cells were transfected with a validated small interfering RNA (siRNA) targeting the HIF-1α sequence, 5′-GCAAUAGACAAGGACAUAATT-3′ (synthesized by Shanghai GenePharmaCo., Ltd. China). Lipofectamine™ 2000 reagent (Invitrogen, USA) was utilized to perform the transfection according to the manufacturer’s protocol, and the cells were incubated at 37 °C for 48 h before analysis. 48 h later, western blot assay was conducted to confirm the gene silencing effects.

### Establishing ROCK1 & ROCK2 overexpressing cells

One day before transfection, 2 × 10^4^ HCC were seeded into 24-well plate. Lenti-virus (Obio, Shanghai, China) was applied to transduce the cells with the corresponding vectors. 24 h later, the transduced cells were diluted with proportion of 1:100 and plated into a 100-mm culture dish. In order to select for cells overexpressing ROCK1&2, the cells were cultured in medium with 5 μg/mL puromycin for 14 days. Clones displaying puromycin resistance and expressing the fluorescent label (Flag) survived and expanded.

As for lentivirus construction, short hairpin RNA (shRNA) for ROCK1 or ROCK2 and the negative control were cloned into the lenti knockdown vector pLenti-U6-shRNA-CMV- EGFP-T2A-Puro to produce Lenti-U6-shRNA (ROCK1 or ROCK2)-CMV- EGFP-T2A-Puro. The core target sequences of the shRNA are presented in Additional file 2: Table S2. Moreover, the full length of ROCK1 or ROCK2 was cloned into the overexpress vector Lenti-CMV-MCS-3FLAG-PGK-Puro H156 to produce pLenti-CMV-(ROCK1 or ROCK2)-3FLAG-PGK-Puro (Obio Technology Co., Ltd., Shanghai, China), the primers for amplification of ROCK1 and 2 are listed in Additional file 2: Table S2. The stable knockdown and overexpression of ROCK1 or ROCK2 was confirmed by Western blotting.

### Immunohistochemical (IHC), CD34/PAS double staining and scoring

The tissue slices were deparaffinized, hydrated according to standard protocols. Antigen retrieval was performed, and unspecific binding sites were blocked using BSA. Then, the slices were incubated within a series of primary antibodies (Additional file 1: Table S1) overnight at 4 °C, and the corresponding secondary antibody was incubated with each slice at 37 °C for 30 min. The color was developed by a 3,3′-diaminobenzidine chromogen (DAB, Gene Tech GTVisionIII Detection Kit, Shanghai, China) solution. All of the slices were counterstained with hematoxylin, dehydrated, and mounted.

Immunohistochemical staining with CD34 was performed on the sections as described above prior to PAS staining. Then, the slides were treated with periodic acid solution for 10 min and rinsed with distilled water for 5 min. In a dark chamber, the slides were submerged in Schiff solution for 15 min at 37 °C. After washing the slides under running water for 20 min, all of the sections were counterstained with hematoxylin, dehydrated, and mounted. VM channels surrounded by HCC cells were positive for PAS staining, but negative for CD34.

At least 10 visual fields for each slides were observed per slide using a double-blind method by two independent investigators according to the staining area and intensity. The percentage of the staining area was scored as follows: 0 (negative staining), 1 (1–25%), 2 (26–50%), 3 (51–75%), 4 (76–100%). Staining intensity was graded as follows: 0 (no staining), 1 (weak staining), 2 (moderate staining), 3 (intense staining). The comprehensive score was calculated as staining percentage multiply intensity. The cases scoring ≥3 were identified as positive expression.

### Western blot analysis

Antibodies dilution in Additional file 1: Table S1. Western blot assay was repeated at least three times. The total proteins were extracted from various groups and electrophoresed by SDS-PAGE. The proteins in the gel were transferred onto a PVDF membrane (Merck-Millipore), and the membrane was blocked with 5% non-fat milk dissolved in TBS containing 1% Tween-20 (TBST) for 1 h at room temperature. The membrane was incubated with a primary antibody at 4 °C overnight, washed with 1% TBST three times, and incubated with an alkaline phosphatase-conjugated secondary antibody for 1 h at room temperature. After washing, the chemiluminescent signal was imaged using a ChemiDoc XRS (Bio-Rad) and quantified using Image J. The experiment was repeated at least three times.

### Real-time qPCR analysis

Total RNA was extracted from cells using Trizol (Invitrogen) and verified using electrophoresis. Then the cDNA was prepared by reverse-transcription kit (Thermo Fisher Scientific). Human GAPDH was used as endogenous control. The primer sequences used in qPCR was listed in Additional file 3: Table S3. The qPCR was performed on a 7900HT Sequence Detection System (ABI, Foster City, CA, USA). The relative expression of the mRNA was calculated by the 2 − ΔΔCt method.

### Matrigel tube formation assay

Tumor cell formation of the capillary structure was tested in vitro as previously described. Briefly, Matrigel was thawed at 4 °C overnight. Then, 100 μl Matrigel (10 mg/ml) was added into each well of a 24-well plate and allowed to solidify at 37 °C for 1 h. The cell suspension with or without treatment in culture medium (2 × 10^4^/ml) was seeded onto the Matrigel-coated 24-well plate, incubated in normoxia or hypoxia for indicated time, photographed, and counted using an inverted light microscope (Nikon, Japan).

### Analysis of RhoA activity

The activity of RhoA was addressed using a biochemical assay that measures the amount of active, GTP-bound RhoA protein (G-LISA RhoA Activation Assay Biochem Kit (absorbance based); Cytoskeleton).

### Statistical analysis

Data were expressed as the mean ± S.E.M of three independent experiments with three biological replicates. Statistical analysis was performed by SPSS V.20.0 Software (SPSS Inc., Chicago, IL, USA). With student’s t test, Chi-squared test and Fisher’s exact test, we analyzed the statistical significance. *P* values < 0.05 (two-tailed) were considered as statistically significant.

## Results

### VM presented in HCC tissues

**Fig. 1:**
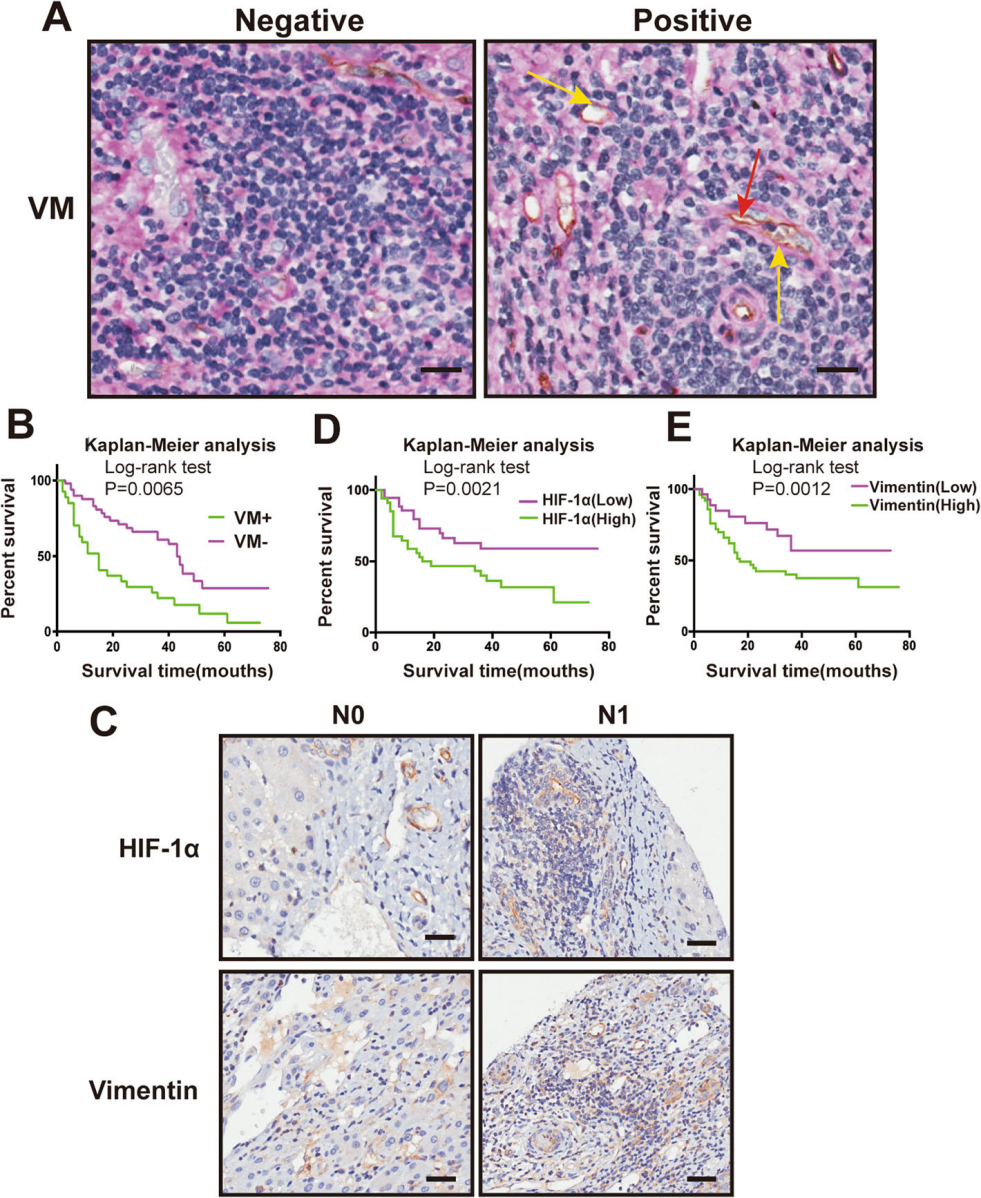
Evidence of VM in HCC tumor tissues and HIF-1α & Vimentin expression contributing to poor prognosis in HCC. **a.** Endothelium-dependent vessels (yellow arrow) exhibited CD34-positive on their luminal surface, and positive reaction to PAS in their wall. VM channels (red arrow) made of HCC cells were positive for PAS staining, but negative for CD34. The ‘Negative’ in left panel represents a region without blood vessels and VM. Original magnification 200 ×. The scales represent 50 μm. **b**. Survival analysis based on VM. **c**. HCC specimens were analyzed by HIF-1α or Vimentin immunohistochemistry associated with no lymph node metastasis (N0) and lymph node metastasis (N1). **d** Survival analysis based on HIF-1α expression. **e** Survival analysis based on Vimentin expression. Original magnification 200 ×. The scales represent 50 μm

CD34-PAS double staining was performed to detect VM. VM was defined as a pattern of blood supply in carcinoma without participation by endothelial cells. Endothelium-dependent vessels exhibited CD34+ on their luminal surface [22]. While, VM channels made of HCC cells were positive for PAS staining, but negative for CD34. As shown in Fig. 1a (yellow arrow indicating blood vessel; red arrow indicates VM channels), VM channels were presence in tumor tissues, but absence in corresponding non-cancerous tissues. Moreover, a Kaplan-Meier assay showed that patients who VM+ tended to have a poor prognosis (*P < 0.05; Fig. 1b).

### Correlated expression of HIF-1α and Vimentin in clinical HCC tissues

To determine whether HIF-1α was involved in VM development, we analyzed HIF-1α and Vimentin expression in 77 pairs of HCC and adjacent non-cancerous specimens. The clinical data in Table 2 showed that HIF-1α was highly expressed in 43 of 77 HCC tissues (55.8%), Vimentin was expressed in 50 of 77 HCC tissues (64.9%). Enhancive HIF-1α and Vimentin expression was correlated with an increase in lymph nodes metastasis (*P < 0.05; Table 1; Fig. 1c). Remarkably, our analysis data illustrated that the prevalence of VM was inextricably linked with the expression of HIF-1α and Vimentin (Table 1). Moreover, a Kaplan-Meier assay showed that patients with high HIF-1α expression or Vimentin expression tended to have a poor prognosis (*P < 0.05; Fig. 1d, e). To verify this hypothesis: HIF-1α and Vimentin were correlated in HCC patients, a correlation analysis data showed that HIF-1α expression was positively correlated with Vimentin expression in HCC patients (*P < 0.05, Table 3). While there is no relationship detected between HIF-1α or Vimentin protein and age, gender, tumor size, invasion depth and distant metastasis (Table 1). Moreover, comparing with other patients, VM positivity is significantly high in HIF-1α and Vimentin double-high patients (***P < 0.0001, Table 4). Altogether, these data showed that both HIF-1α and Vimentin were upregulated, and they could synergistically promote HCC progression and VM formation.

**Table 2:** Protein expression of HIF-1α and Vimentin in HCC cancer tissues and adjacent normal tissues

Tissue sample	No. of patients	HIF-1α		*P*-value	Vimentin		*P*-value
		Low (%)	High (%)		Low (%)	High (%)	
Tumor	77	34(44.2)	43(55.8)	0.000*	27(35.1)	50(64.9)	0.000*
Non-cancerous tissues	77	66(85.7)	11(14.3)		64 (83.1)	13(16.9)	

HIF-1α and Vimentin expression was measured in tumor and non-cancerous tissues. Both HIF-1α and Vimentin was higher in tumor tissues compared with non-cancerous tissues. Data were analyzed using the Chi-squared test

*P < 0.05 indicates statistical significance

**Table 3:** Correlation analysis between HIF-1α and Vimentin protein expression in HCC

Tumor tissue sample	HIF-1α		Correlation coefficient	*p*-value
	Low	High		
Vimentin Low	7	20	−0.270	0.018*
Vimentin High	27	23		

*P < 0.05 indicates statistical significance

**Table 4:** Correlation analysis between VM and HIF-1α, Vimentin protein expression in HCC

Tumor tissue sample	VM		Correlation coefficient	*p*-value
	Positive	Negative		
Double High	18	5	0.5087	< 0.0001***
All others	9	45		

*P < 0.05 indicates statistical significance

### Hypoxia regulated rho/ROCKs and Rac1/PAKs expression

**Fig. 2:**
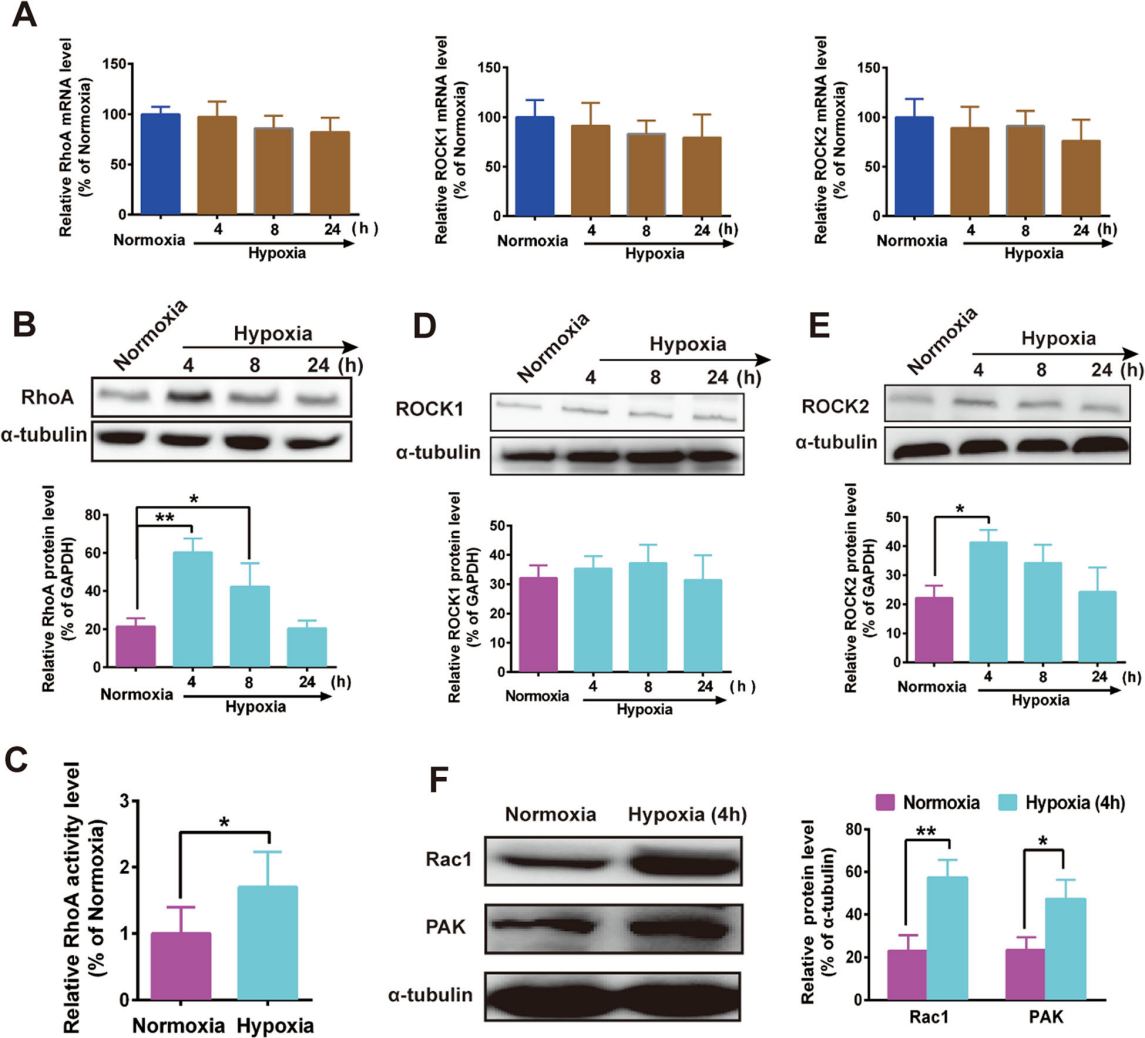
Hypoxia upregulated RhoA and ROCK2 protein expression. **a** Effect of hypoxia on RhoA, ROCK1 and ROCK2 mRNA for 24 h. Lack of significant effect of hypoxia (4, 8 and 24 h) on RhoA/ROCK mRNA. **b** Time course of the effects of hypoxia on RhoA protein analysed by Western blot. **c** RhoA activity measured with a biochemical assay. Time course of the effects of hypoxia on ROCK1 (**d**) and ROCK2 (**e**) proteins, quantitated using Image J. ROCK2 protein was markedly increased after 4 h in hypoxia. **f** Effects of hypoxia on Rac1 and PAK proteins, quantitated using Image J. Normoxia condition as positive control. The data are expressed as the mean ± S.E. of three independent experiments. *P < 0.05; **P < 0.01 vs normoxia. h = hours

To query whether the RhoA/ROCK pathway is regulated by hypoxia, a time-dependent study of gene and protein expression assay was performed to compare levels of RhoA, ROCK1 or ROCK2 expression under hypoxia conditions. Exposure of MHCC97H to hypoxia for 4–24 h did not produce any significant effect on RhoA mRNA (Fig. 2a, left panel), but increased its protein level between 4 and 8 h (Fig. 2b). To assess the activation state of RhoA, we used an ELISA-based assay that measures the level of GTP-bound RhoA. Exposure of MHCC97H to 1% O_2_ (4 h) induced a significant increase in RhoA activity (Fig. 2c). Similar to RhoA, exposure MHCC97H to hypoxia did not affect the mRNA level of both ROCK1 and ROCK2 (Fig. 2a, middle and right panels). Interesting, hypoxia upregulated ROCK2 protein level for 4 h and 8 h, but did not affect the expression of ROCK1 (Fig. 2d, e). These results indicate that being exposed to 1% O_2_ for 4 h showed the maximal synergistic effect. As a result, we used an incubation time of 4 h in the following experiments. Similar to RhoA/ROCKs, exposure of MHCC97H to 1% O_2_ (4 h) induced a significant increase expression of Rac1 and PAK (Fig. 2f). Taken together, those data above provided evidence for hypoxia-dependent activity of RhoA/ROCK2 and Rac1/PAK signaling in HCC.

### RhoA/ROCKs and Rac1/PAK act upstream of HIF-1α

**Fig. 3:**
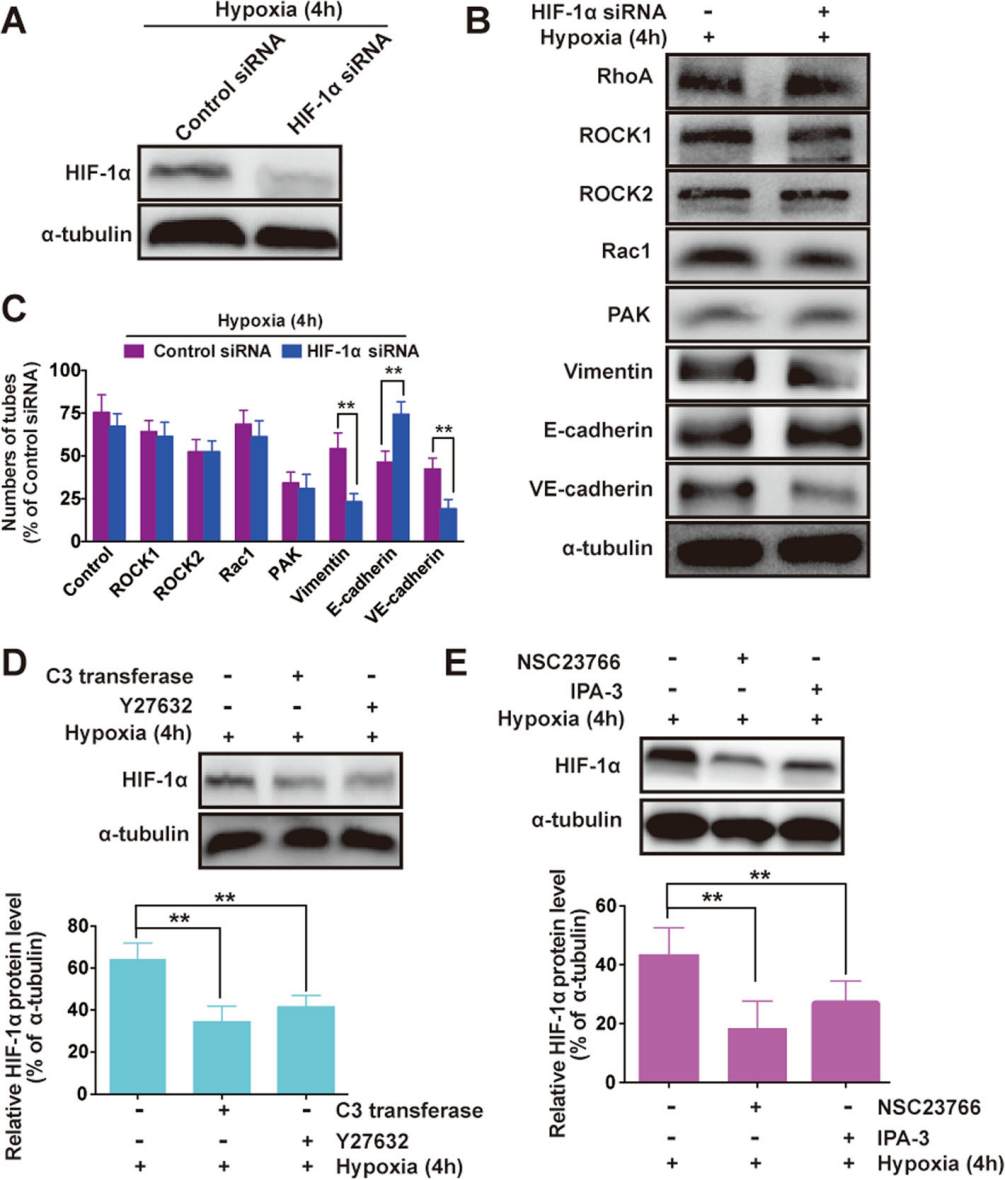
RhoA/ROCKs and Rac1/PAK act upstream of HIF-1α. **a** Western blot analysis for the expression of HIF-1α in MHCC97H cell lines transfected with control siRNA or HIF-1α siRNA. **b** Western blot analysis for RhoA/ROCK, Rac1/PAK, EMT (Vimentin and E-cadherin) and VE-cadherin (a biomarker of VM), in MHCC97H cells transfected with control siRNA or HIF-1α siRNA. The cells were exposed to hypoxia for 4 h. **c** Quantitated using Image J *P < 0.05; **P < 0.01 vs. control siRNA. Western blot analysis for HIF-1α expression when treated with (**d**) C3 transferase or Y27632, and (**e**) NSC23766 or IPA-3, Quantitated using Image J. The data are expressed as the mean ± S.E. of three independent experiments. **P < 0.01 vs. Hypoxia (4 h). h = hours

HIF-1α is the key regulatory component, because it is rapidly degraded in normoxic conditions but stabilized and activated in hypoxia [23, 24]. To test if HIF-1α stabilization affects RhoA/ROCKs, Rac1/PAK, EMT and VM markers activity, knockdown HIF-1α in MHCC97H cells was established. In cells transfected with HIF-1α siRNA, the HIF-1α protein expression was intensely inhibited compared with that in control siRNA transfected cells (Fig. 3a). Inhibition of HIF-1α did not affect RhoA/ROCKs and Rac1/PAK activity exposure of MHCC97H to 1% O_2_ (4 h) (Fig. 3b, c). To examined whether RhoA/ROCKs and Rac1/PAK influenced HIF-1α in HCC, the MHCC97H cells were treated with exoenzyme C3 (800 ng/ml, RhoA inhibitor), Y27632 (50 μM, ROCK inhibitor), NSC23766 (50 μM, Rac1 inhibitor) or IPA-3 (10 μM, PAK inhibitor) in hypoxia. There were significant decline in HIF-1α expression caused by all inhibitors (Fig. 3d, e), which was similar to other research [25]. These data indicate that RhoA/ROCKs and Rac1/PAK proteins participate in the regulation of HIF-1α in hypoxia, but that stabilization of HIF-1α does not affect their activity, suggesting that RhoA/ROCKs and Rac1/PAK act upstream of HIF-1α. As shown in Fig. 3b, HIF-1α siRNA transfection resulted in significant Vimentin and VE-cadherin downregulation, E-cadherin upregulation (**P < 0.01 vs. Control siRNA, Fig. 3c), indicating HIF-1α is involved in EMT and VM formation.

### HIF-1α-induced EMT and VM is rho/ROCK and Rac1/PAK dependent

**Fig. 4:**
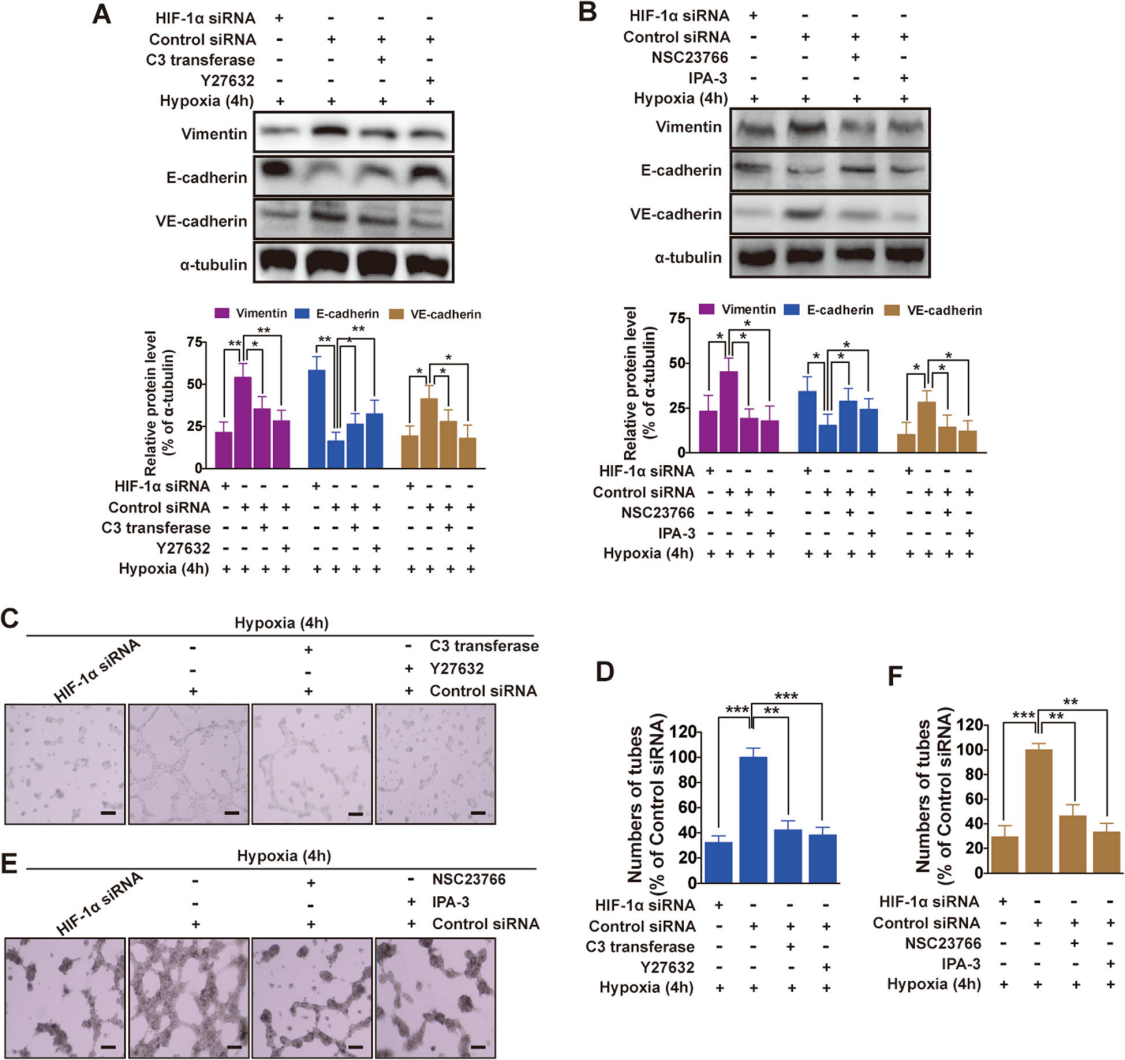
The effect of RhoA/ROCK and Rac1/PAK on VM formation via HIF-1α. Western blot analysis for the expression of Vimentin and E-cadherin and VE-cadherin in HIF-1α siRNA cell treated with (**a**) C3 transferase or Y27632, and (**b**) NSC23766 or IPA-3, Quantitated using Image J. Effects of (**c**) RhoA/ROCK and (**e**) Rac1/PAK on the tubular structure formation activity by 3D culture. Quantitative analysis of the mean number of tube-like structures formed from six randomly chosen areas in 3D cultures using ImageJ. Control siRNA as positive control. The data are expressed as the mean ± S.E. of three independent experiments. Original magnification, 100 ×. The scales represent 50 μm. *P < 0.05; **P < 0.01; ***P < 0.001 vs. Control siRNA. h = hours

To detect whether Rho/ROCK had an inhibitory effect on HIF-1α-mediated neovascularization in hypoxia, we observed the morphology of cell seizure activity in culture medium containing exoenzyme C3, Y27632, NSC23766 or IPA-3, using Control siRNA as the positive control. Exposed to hypoxia (4 h), HIF-1α siRNA transfection resulted in significant Vimentin and VE-cadherin downregulation, E-cadherin upregulation (*P < 0.05, **P < 0.01 vs. Control siRNA, Fig. 4a, b), indicating HIF-1α induces EMT and VM. All inhibitors reversed the high expression induced by HIF-1α (*P < 0.05, **P < 0.01 vs. Control siRNA, Fig. 4a, b). As shown in Fig. 4c and e, being exposed to hypoxia for 4 h, HIF-1α silencing in MHCC97H cells inhibited VM tubes in Matrigel, while control siRNA (HIF-1α normal expression) facilitated VM development, which provided the first evidence addressing the importance of HIF-1α in channel formation. Compared with control siRNA, exoenzyme C3, Y27632, NSC23766 or IPA-3 treatment triggered disappearance of these structures (Fig. 4d, f), suggesting that blockage of Rho/ROCK and Rac1/PAK vanished VM formation through reducing HIF-1α activity. Altogether, those data indicated HIF-1α is involved in EMT and VM formation in RhoA/ROCK and Rac1/PAK dependent manner exposed to hypoxia.

### PAK and ROCK2 facilitated VM by EMT via p-Vimentin (Ser 72 and 56)

**Fig. 5:**
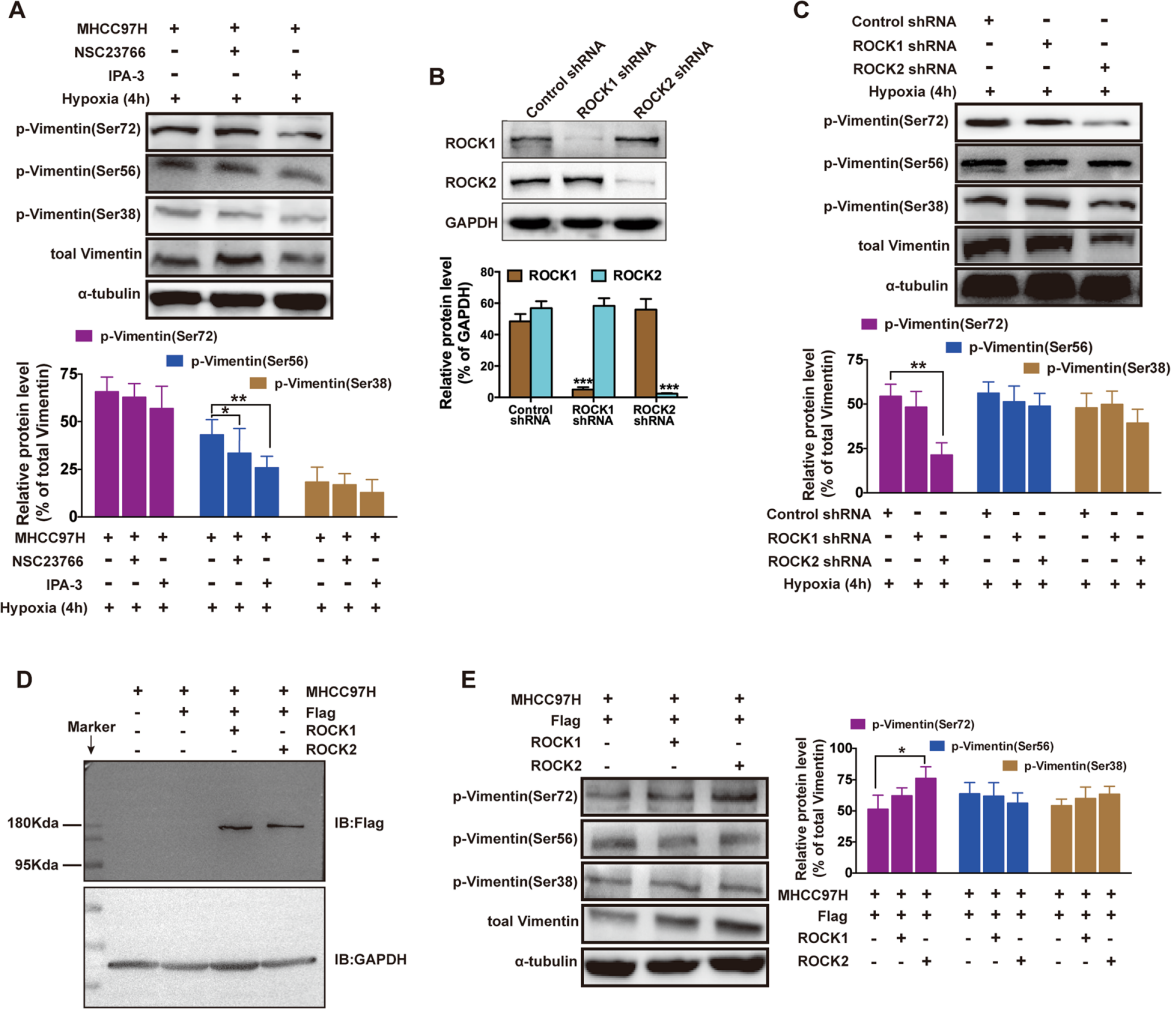
ROCK2 and PAK induced phosphorylation of Vimentin (Ser72 and Ser56). **a** Western blot analysis for p-Vimentin (Ser72, 56 and 38) expression regulated by Rac1/PAK inhibition. **P < 0.01 vs. Hypoxia (4 h). **b, d** Assessing ROCK1 or ROCK2 stably expressing cells by Western blot analysis. **c, e** Western blot analysis for p-Vimentin (Ser72, 56 and 38) expression regulated by knockdown ROCK (**c**) or overexpressed ROCK (**e**). The data are expressed as the mean ± S.E. of three independent experiments. **P < 0.01 vs. Control siRNA, or **P < 0.01 vs. Flag

To investigate the mechanism of PAK and ROCK2 on VM formation, a quantitative protein analysis of Vimentin, Ser72-, Ser56- and Ser38-phosphorylated Vimentin were performed with western blotting. As shown in Fig. 5a, compared to the control, Ser56-phosphorylated Vimentin levels (not Ser-72 or Ser-38) significantly decreased in IPA-3 group, suggesting the potential role of Rac1/PAK on EMT subjected to hypoxia.

To further demonstrate the potential role of ROCK1 or ROCK2 on EMT in hypoxia, ROCK1 and ROCK2 stably expressing cells were established. As shown in Fig. 5b and d, both ROCK1 and ROCK2 were present at detectable levels, indicating that no cross-reactivity occurred between the two constructs.

ROCK is a key serine/threonine kinase responsible for the phosphorylation and subsequent rearrangement of Vimentin [26]. As shown in Fig. 5c, compared to Control shRNA, Ser72-phosphorylated Vimentin levels significantly decreased in ROCK2 shRNA group, not in ROCK1 shRNA, indicating the dynamic changes in Vimentin phosphorylation at Ser72 may reflect the fact that the level of phosphorylated Vimentin is determined by the activation level of ROCK2. The same result appeared in the over-expression of ROCK2 group (Fig. 5e). Strikingly, there was no prominent ROCK-dependent fluctuations of Ser38-phosphorylated Vimentin with fluctuations of RhoA/ROCK or Rac1/PAK. These data suggested that higher active ROCK and PAK expression with the potential of VM formation, may promote Vimentin phosphorylation at Ser72 and Ser56, which may induce the rearrangement of Vimentin caused by ROCK2 and PAK.

## Discussion

Tumor hypoxia was a well-known microenvironment occurring for additional growth [27]. HIF-1 is a heterodimer complex which is consisted of two basic helix-loop-helix (bHLH) transcription factors (HIF-1α and HIF-1β), and it plays a role in responding to hypoxia [28, 29]. In the hypoxic states, HIF-1α is stable and it translocates from the cytoplasm to the nucleus to dimerize with HIF-1β then activate downstream target genes which accelerate cancer progression and promote tumor aggressiveness, including VM [5]. The significant role of HIF-1α in tumor development was first identified due to its abundant overexpression in a broad range of tumor types and its participation in critical aspects of tumor initiation and progression. In our study, HIF-1α expression was correlated with a higher rate in lymph nodes metastasis and VM presentation, which is an independent prognostic marker for HCC (Table 2; Fig. 1). This observation is in accordance with previous work by Li, et al., who reported particularly HIF-1α -induced vasculogenic mimicry formation in human colorectal cancer cells [30].

RhoA and Rac1, both of the Rho family of small GTPases [31], together with Rho kinase was key regulator of both cell adhesion and the cytoskeleton. Our previous research mentioned that blocking ROCK would inhibit migration, invasion and VM formation [4]. As a transcription factor, HIF-1α could affect cell plasticity from several dimensions [32], showed that HIF-1α may induce VM, similar to our results: HIF-1α silencing inhibited VM tubes in Matrigel. In light of these results, we were interested in identifying the upstream events that trigger the upregulation of RhoA/ROCK and Rac1/PAK in VM formation responding to hypoxia. Notably, we mentioned that RhoA may not be essential for the VM process in normoxia condition [4], contrary to our present research. To our knowledge, the relationship between hypoxia and RhoA is very complicated, even contrary [33, 34]. Rho-GTPases, which are regulated by VEGF, play a crucial role in cancer progression [35, 36]. VEGF-A expression was mediated via both HIF-1α-dependent and -independent mechanisms [37, 38]. Those reports indicated that exposure to hypoxia induces a significant increase in RhoA activity, similar our present report. We found that hypoxia upregulated RhoA/ROCK2 and Rac1/PAK protein level, but did not affect the expression of ROCK1. Recent reports have established some cross-talk between the HIF, RhoA/ROCK and Rac1/PAK pathways. It has been shown that, in some cell types, RhoA is responsible for HIF-1α mRNA and/or protein induction in low oxygen conditions [39, 40]. Our results above demonstrated that HIF-1α did not regulate RhoA/ROCK and Rac1/PAK activity, but inhibition of RhoA and Rac1 activity reduces the hypoxic stabilization of HIF-1α, indicating that the small monomeric G protein is involved in HIF-1α regulation in HCC. In addition, exoenzyme C3, Y27632, NSC23766 or IPA-3 treatment vanished VM formation, confirming the contribution of RhoA/ROCK and Rac1/PAK to VM formation in hypoxia. In summary, our results show that hypoxia induces an increase in RhoA and Rac1 activity and stabilizes HIF-1α protein, suggesting the existence of cross-talk between RhoA, Rac1 and HIF pathways.

The FAK-Rac-PAK pathway also responds to mechanical signals and is important in Vimentin network disassembly. FAK phosphorylation facilitates Rac activation [41], then activates PAK and alters cytoskeletal dynamics [42]. PAK phosphorylates Vimentin [43] to cause network disassembly. We show that PAK inhibition decrease p-Vimentin (Ser 56) protein level, but did not affect the expression of p-Vimentin (Ser 72 or 38) (Fig. 5a), similar to other report [44]. The results of Rac1 affecting p-Vimentin (Ser 56) protein could be caused by the loss of PAK activation and consequent unstabilization of the Vimentin. ROCKs consist of two isoforms: ROCK1 and ROCK2. ROCK1(−/−) mice display failure of the eyelid and ventral body wall closure and die soon after birth [45], while ROCK2(−/−) mice experience embryonic lethality due to intrauterine growth retardation and placental dysfunction [46], suggesting that the regulation and signaling of these two proteins may be divergent to a measurable degree. Hypoxia sensitized the RhoA/ROCK2 signaling pathway in HCC (Fig. 2b and e), then enhancement of p-Vimentin (Ser 72) was observed in Flag-ROCK2 but not in Flag-ROCK1 cell lines (Fig. 5c and e). However, ROCK1 was upregulated in neonatal rat ventricular myocyte subjected to hypoxia [47]. These results indicated that the roles of ROCK1 were different from those of ROCK2 in hypoxic condition and various pathology [48].

EMT was closely related to an aggressive tumor phenotype in HCC [49]. Here, stable knockdown of HIF-1α in MHCC97H cell lines increased E-cadherin and downregulated Vimentin protein expression (Fig. 3b). So the HIF-1α could induce EMT, which brought out cell plasticity. HIF-1α was a critical parameter in VM [50], VE-cadherin was the first reported biomarker of VM [3]. In this study, inhibition of VE-cadherin followed HIF-1α siRNA transfection (Fig. 3b), concurring with 3D culture assay (Fig. 4c and d). Our data suggested that VM was observed after EMT in vitro.

**Fig. 6:**
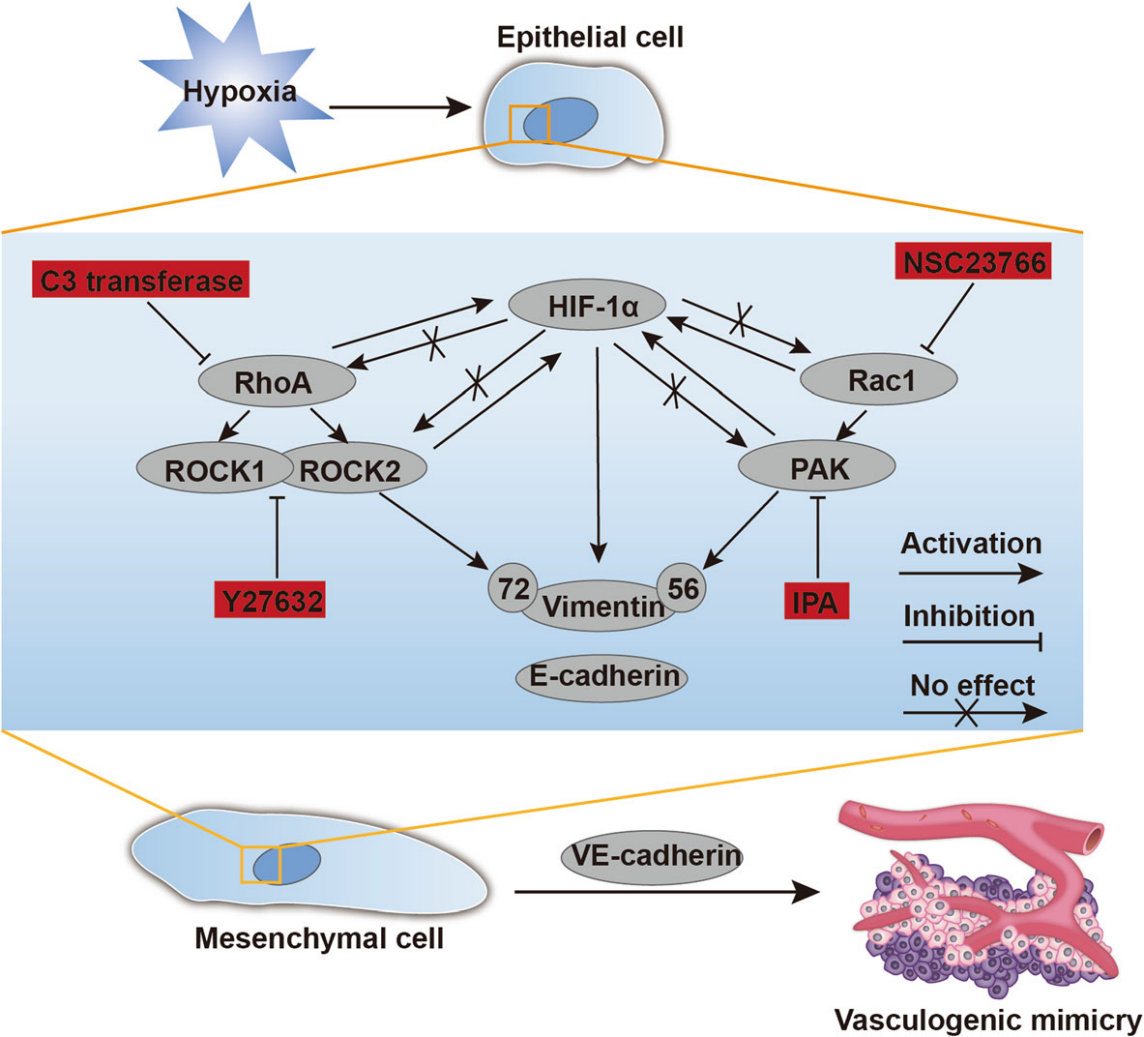
Schematic overview summarizing the functional significance and regulation of RhoA/ROCK, Rac1/PAK and HIF-1α exposed to hypoxia in HCC. Hypoxia triggers the upregulation of RhoA/ROCK2 and Rac1/PAK, subsequently modulates HIF-1α stabilization, leading to elevated Vimentin expression with phosphorylation at Ser72 and Ser56, which finally induces VM development via promoting the secretion of VE-cadherin accompany occurrences of EMT (Epithelial cell transform to Mesenchymal cell) in hypoxia

Taken together, we showed that HIF-1α and Vimentin contributed to a poor prognosis in HCC patients. Then, we expounded that hypoxia could enhance RhoA/ROCK and Rac1/PAK expression, further regulate HIF-1α expression. Ultimately, RhoA/ROCK and Rac1/PAK induced VM formation by HIF-1α stabilization and EMT with p-Vimentin (Ser72 and 56) activated (Fig. 6).

## Conclusions

This study showed that HIF-1α and Vimentin contributed to a poor prognosis in HCC patients. Hypoxia could enhance RhoA/ROCK and Rac1/PAK expression, further regulate HIF-1α expression. Ultimately, RhoA/ROCK and Rac1/PAK induced VM formation by HIF-1α stabilization and EMT with p-Vimentin (Ser72 and 56) activated.

## Supplementary information

**Additional file 1: Table S1.** The information of antibodies used in this study

**Additional file 2: Table S2.** shRNA Construct Sequences

**Additional file 3: Table S3.** Sequences of primers

## Data Availability

The datasets used and analyzed during the present study are available from the corresponding author upon reasonable request.

## Abbreviations

EMT: Epithelial-mesenchymal transition
HCC: Hepatocellular carcinoma
HIF-1α: Hypoxia inducible factor-1α
IHC: Immunohistochemical
PAK: p21-activated kinase
PAS: Periodic acid-Schiff
ROCK: Rho kinases
shRNA: Short hairpin RNA
siRNA: Small interfering RNA
VM: Vasculogenic mimicry
