# Supplementary material for: Hypoxic induction of vasculogenic mimicry in hepatocellular carcinoma: role of HIF-1 α, RhoA/ROCK and Rac1/PAK signaling
Source: BMC Cancer. 2020 Jan 13;20:32. doi: 10.1186/s12885-019-6501-8 (PMC6958789; doi:10.1186/s12885-019-6501-8)
Supplement: Supplementary file 1 — Additional file 1: Table S1. The information of antibodies used in this study [file 12885_2019_6501_MOESM1_ESM.docx]

**Additional file 1: Table S1. The information of antibodies used in this study**

| Antibodies | Company | Clone | Product Number | Dilution | |
| --- | --- | --- | --- | --- | --- |
|  |  |  |  | IHC | Western blot |
| CD34 | Santa Cruz | Mouse | sc7324 | 1:100 | / |
| HIF-1α | Abcam | Rabbit | ab16066 | / | 1:1000 |
| RhoA | Abcam | Rabbit | ab187027 | / | 1:1000 |
| ROCK1 | Abcam | Rabbit | ab45171 | / | 1:2000 |
| ROCK2 | Abcam | Rabbit | ab71598 | / | 1:2000 |
| VE-Cadherin | Cell Signaling | Rabbit | 2500 | / | 1:200 |
| Vimentin | Abcam | Rabbit | ab52942 | 1:200 | 1:1000 |
| E-cadherin | Abcam | Rabbit | ab1416 | / | 1:1000 |
| Rac1 | Abcom | Mouse | ab33186 | / | 1:1000 |
| PAK | Abcom | Rabbit | ab223849 | / | 1:1000 |
| p-Vimentin(Ser72) | abcom | Rabbit | ab52944 | / | 1:10000 |
| p-Vimentin(Ser56) | Abcam | Rabbit | ab217673 | / | 1:1000 |
| p-Vimentin(Ser38) | Abcam | Rabbit | ab52942 | / | 1:100000 |
