# Supplementary material for: Hypoxic induction of vasculogenic mimicry in hepatocellular carcinoma: role of HIF-1 α, RhoA/ROCK and Rac1/PAK signaling
Source: BMC Cancer. 2020 Jan 13;20:32. doi: 10.1186/s12885-019-6501-8 (PMC6958789; doi:10.1186/s12885-019-6501-8)
Supplement: Supplementary file 2 — Additional file 2: Table S2. shRNA Construct Sequences [file 12885_2019_6501_MOESM2_ESM.docx]

**Additional file 2: Table S2. shRNA Construct Sequences**

| Gene | Sequence |
| --- | --- |
| ROCK1 | 1) 5’-GGTATATGCTATGAAGCTT-3’ |
|  | 2) 5’- GCACCAGTTGTACCCGATT-3’ |
|  | 3) 5’- GCATTTGAGGCAGACAATT-3’ |
|  | 4) 5’-GCAATATTTCTCGACACTT-3’ |
| ROCK2 | 1) 5’-GGTTTATGCTATGAAGCTT-3’ |
|  | 2) 5’-GCAGCAATTTCGATGACAT-3’ |
|  | 3) 5’-GCAACTGGCTCGTTCAATT-3’ |
|  | 4) 5’-GCACCTTGCAAAGTATATT-3’ |
| Control | 5’-TTCTCCGAACGTGTCACGT-3’ |

**Primers for amplification**

| **Gene** | **Primer** |
| --- | --- |
| ROCK1 | 1) 5’-CGCAAATGGGCGGTAGGCGTG -3’ |
|  | 2) 5’- CAGCGGGGCTGCTAAAGCGCATGC -3’ |
| ROCK2 | 1) 5’- CGCAAATGGGCGGTAGGCGTG -3’ |
|  | 2) 5’- CAGCGGGGCTGCTAAAGCGCATGC -3’ |
| Control |  |
