# Supplementary material for: Hypoxic induction of vasculogenic mimicry in hepatocellular carcinoma: role of HIF-1 α, RhoA/ROCK and Rac1/PAK signaling
Source: BMC Cancer. 2020 Jan 13;20:32. doi: 10.1186/s12885-019-6501-8 (PMC6958789; doi:10.1186/s12885-019-6501-8)
Supplement: Supplementary file 3 — Additional file 3: Table S3. Sequences of primers [file 12885_2019_6501_MOESM3_ESM.docx]

**Additional file 3: Table S3. Sequences of primers**

| Gene | | Forward Primer (5’-3’) | Reverse Primer (5’-3’) | |
| --- | --- | --- | --- | --- |
| RhoA | GGATCTTCGGAATGATGAGCA | | TGTTTGCCATATCTCTGCCTTCT |  |
| ROCK1 | TATGAAGTAGTAAAGGTAATCGGCAGAG | | CTGGTGGATTTATGCCTTACCAA |  |
| ROCK2 | AATCAAATCAGCATCCTTCTTTAAGAAT | | CTGGAGCTGCCGTCTCTCTTAT |  |

All the sequences were based on the published data on the National Center for Biotechnology Information followed by the accession number.
